# Established and Novel Indications for Left Ventricular Unloading—When and for Whom?

**DOI:** 10.3390/jcdd13100528

**Published:** 2026-10-20

**Authors:** Andreas Schäfer, Tobias J. Pfeffer, Bastian Schmack, Johann Bauersachs

**Affiliations:** 1 Department of Cardiology and Angiology, Hannover Medical School, Carl-Neuberg-Str. 1, D-30625 Hannover, Germany; pfeffer.tobias.j@mh-hannover.de (T.J.P.); bauersachs.johann@mh-hannover.de (J.B.); 2 Clinical Department of Cardiothoracic, Transplantation and Vascular Surgery, Hannover Medical School, , Carl-Neuberg-Str. 1, D-30625 Hannover, Germany; schmack.bastian@mh-hannover.de

**Keywords:** mechanical circulatory support, microaxial flow pump, cardiogenic shock

## Abstract

Temporary mechanical circulatory support devices are increasingly used to stabilise haemodynamics in patients with compromised cardiac output. While devices vary regarding indications and haemodynamic effects, only left ventricular (LV) unloading by microaxial flow pumps in a specific subset of patients with acute ST-segment elevation myocardial infarction-associated cardiogenic shock (AMI-CS) has shown a reduction in mortality. This review elucidates on this evidence-based indication for LV unloading, namely patients with AMI-CS fulfilling the Danish-German (DanGer)-Shock trial’s inclusion and exclusion criteria. Furthermore, the review discusses cardiogenic shock scenarios encountered in everyday clinical practice beyond the trial population. It further extends beyond that, discussing clinical scenarios such as protected percutaneous coronary intervention including critical appraisal of recent trials, the negative results of unloading trials in AMI, as well as the settings of protected ventricular tachycardia ablation, LV unloading in veno-arterial extracorporeal membrane oxygenation (vaECMO) cases, escalated support with Impella 5.5 for postcardiotomy patients and as a bridge-to-decision or bridge-to-LV assist device (LVAD), and emerging indications such as protected cardiac as well as non-cardiac surgery.

## 1. Introduction

Temporary mechanical circulatory support (MCS) encompasses a range of devices used to stabilise haemodynamics in patients with compromised cardiac output. Although these devices differ in their haemodynamic properties, this article focuses primarily on the group of devices that have recently received favourable recommendations in clinical guidelines [1], based on the first randomised controlled trial (RCT) to demonstrate a mortality benefit in cardiogenic shock [2]: the Impella^®^ family of microaxial flow pumps (mAFPs), which are used to unload the left ventricle (LV). These devices have been available for almost two decades and are increasingly being used for a variety of indications. However, the evidence supporting their use varies across indications, some of which are established while others are emerging on the basis of pathophysiological considerations. The available evidence also differs in terms of the number and outcomes of RCTs, with some trials demonstrating benefit and others yielding neutral results; moreover, trial progress has varied, with some studies completed as planned and others terminated prematurely [3,4,5,6].

Initially, mAFPs were used to provide haemodynamic support during percutaneous coronary intervention (PCI) in high-risk patients with severely impaired LV function. In these patients, repeated episodes of ischaemia caused by balloon inflation during PCI of the last remaining patent or dominant coronary artery were thought to compromise haemodynamic stability [7]. Given their ability to stabilise haemodynamics, mAFPs were subsequently used more broadly in patients with cardiogenic shock (CS), particularly those with infarct-related CS. The aim was to stabilise patients sufficiently to permit comprehensive PCI and revascularisation, thereby potentially contributing to shock resolution and reducing mortality [8]. Although RCTs investigating mAFP use during high-risk PCI have not demonstrated a significant benefit in their primary endpoints, the Danish-German (DanGer)-Shock trial was the first RCT to demonstrate a statistically significant mortality benefit with a specific MCS device in a defined clinical indication, namely acute myocardial infarction-related cardiogenic shock (AMI-CS). Furthermore, the use of mAFPs is increasing in advanced heart failure (HF) and in patients with severe chronic HF undergoing cardiac or non-cardiac surgery. An overview of the available mAFPs is provided in Table 1.

## 2. Indications for LV Unloading

**Figure 1:**
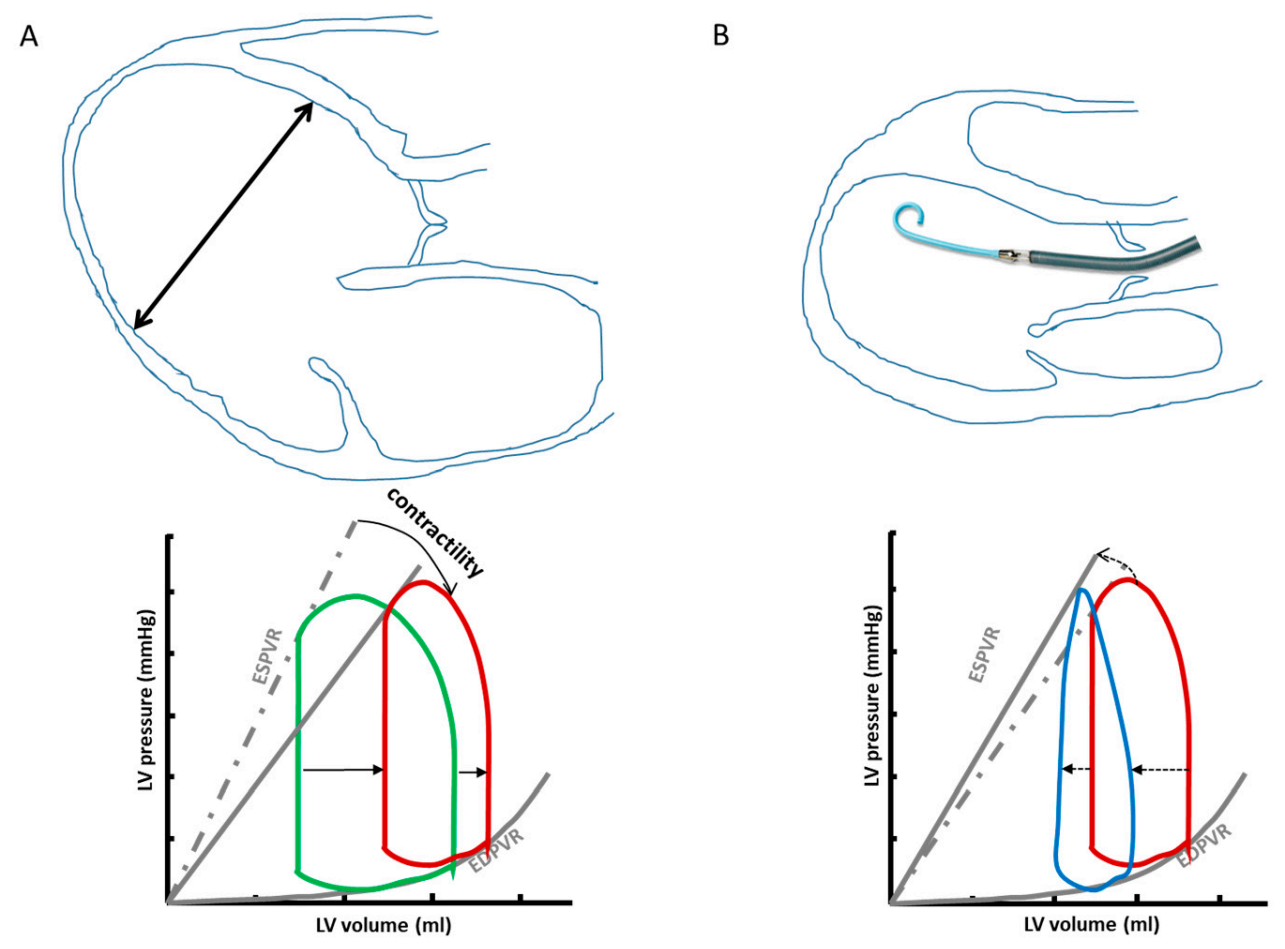
Dimensions and haemodynamics of the left ventricle (LV) (**A**) during overload and distension and (**B**) during LV unloading with microaxial flow-pumps (mAFP). The upper panel indicates LV dilation (left) and placement of an mAFP (right). The lower panel indicates the respective change in the LV pressure–volume loop from physiologic (green) to LV overloading (red) and the reversal by LV unloading using an mAFP (blue). EDPVR = end-diastolic pressure–volume relation; ESPVR = end-systolic pressure–volume relation.

LV unloading using mAFPs is an attractive therapeutic concept in clinical scenarios characterised by LV overload and decompensation. Almost any clinical condition associated with a substantial loss of myocardial contractility can ultimately lead to LV distension and overload, depending on the severity of dysfunction and the ability of the remaining functional myocardium to compensate for the failing myocardium. mAFPs actively unload the compromised LV, thereby reducing LV dilatation and congestion while simultaneously providing haemodynamic support by contributing directly to systemic cardiac output [7,9]. Clinical scenarios in which LV unloading may be considered include AMI-CS [2,8,10], acute myocarditis [11,12], acute cardiomyopathies such as Tako-Tsubo syndrome [13,14] or peripartum cardiomyopathy [15,16], acute decompensation of dilated cardiomyopathy [17], and acute deterioration in patients with pre-existing severe LV dysfunction due to additional ischaemia during PCI [18,19] or cardiac surgery [20]. Other potential indications include prolonged arrhythmia during LV ablation procedures [21] and haemodynamic deterioration during general anaesthesia for non-cardiac surgery [22,23]. The haemodynamic rationale for unloading a decompensated LV is illustrated in Figure 1.

### 2.1. Protected High-Risk Percutaneous Coronary Intervention

The elective use of mAFPs during protected high-risk PCI was initially investigated in the PROTECT II trial [19]. The trial evaluated the Impella^®^ 2.5 in patients with impaired LV function undergoing PCI. High-risk, non-emergent PCI was defined as PCI in adults with an LV ejection fraction (LVEF) of ≤35% undergoing intervention on an unprotected left main coronary artery or the last remaining patent coronary artery, or an LVEF of ≤30% in patients undergoing multivessel PCI for three-vessel disease. The trial was designed and powered to enrol 654 patients but was terminated for presumed futility after 448 patients had been randomised. The primary endpoint was a composite of all-cause death, myocardial infarction, stroke or transient ischaemic attack, any repeat revascularisation, cardiac or vascular surgery, acute renal insufficiency, severe intraprocedural hypotension requiring treatment, cardiopulmonary resuscitation, ventricular tachycardia requiring cardioversion, aortic insufficiency, and angiographic failure of PCI at 30 days. The primary endpoint was not significantly reduced. Consequently, the trial did not provide robust evidence to support the routine use of mAFPs during high-risk PCI in patients with impaired LVEF. An exploratory per-protocol analysis nevertheless suggested a reduction in symptoms and an improvement in LVEF at 90 days [19,24]. A lower incidence of acute kidney injury was also reported in patients undergoing mAFP-supported high-risk PCI [25]. Given the neutral primary endpoint, however, these findings should be considered hypothesis-generating only. Despite the absence of definitive evidence of benefit, the use of mAFPs during PCI procedures has increased in clinical practice, including in patients who do not fully meet the PROTECT II inclusion criteria [26]. Until further evidence becomes available, international consensus has supported considering mAFPs in highly symptomatic patients with severe LV dysfunction whose symptoms persist despite optimal medical therapy and who are not candidates for surgical revascularisation [18]. One limitation of the original RCT was the use of Impella 2.5, which provides relative limited support of up to 2.5 L/min. This device was subsequently superseded in many clinical settings by the Impella CP, which provides approximately 3.5–4.0 L/min of flow. More recently, another manufacturer conducted the SUPPORT I trial, a prospective, single-arm feasibility study involving 15 patients undergoing protected high-risk PCI with the Supira percutaneous ventricular assist device (pVAD). This device provides up to 5.5 L/min of flow through a 10F femoral access [27].

Another major trial in the field of protected high-risk PCI was CHIP-BCIS3, which did not demonstrate a reduction in major adverse clinical outcomes with mAFP-supported PCI [28]. Although the trial was well conducted, it used a win-ratio analysis rather than a conventional composite clinical endpoint. Several aspects of the enrolled population warrant consideration: 23% of patients had no angina at baseline, more than 25% were scheduled for a retrograde approach to treat a chronic total occlusion, and 63% were not scheduled for elective procedures [28]. Furthermore, the investigators reported a mean residual syntax score of 14 when the trial was initially presented during ACC2026, suggesting that substantial residual coronary disease remained after PCI in many participants. The level of device-related experience at participating centres was not reported. In several respects, the study population differed substantially from the considered appropriate candidates in the previous European consensus document [18], potentially limiting the applicability of the findings to the intended target population. The neutral overall result of CHIP-BCIS3 was driven by high all-cause mortality [28], an outcome that may reflect the substantial burden of underlying disease in this population rather than complications directly attributable to the coronary procedure [29].

The ongoing PROTECT-IV trial may provide further clarity regarding the role of mAFPs in protected high-risk PCI. PROTECT-IV is comparing PCI with and without mAFP support in high-risk patients with complex coronary artery disease and reduced LVEF. The primary endpoint is a composite of all-cause death, stroke, implantation of a durable LV assist device, heart transplantation, myocardial infarction, or hospitalisation for cardiovascular causes at three years. The trial plans to enrol 1252 patients with an LVEF of ≤40% undergoing complex PCI, including interventions involving complex multivessel disease, the last remaining patent coronary artery, or left main coronary artery disease [30].

**Figure 2:**
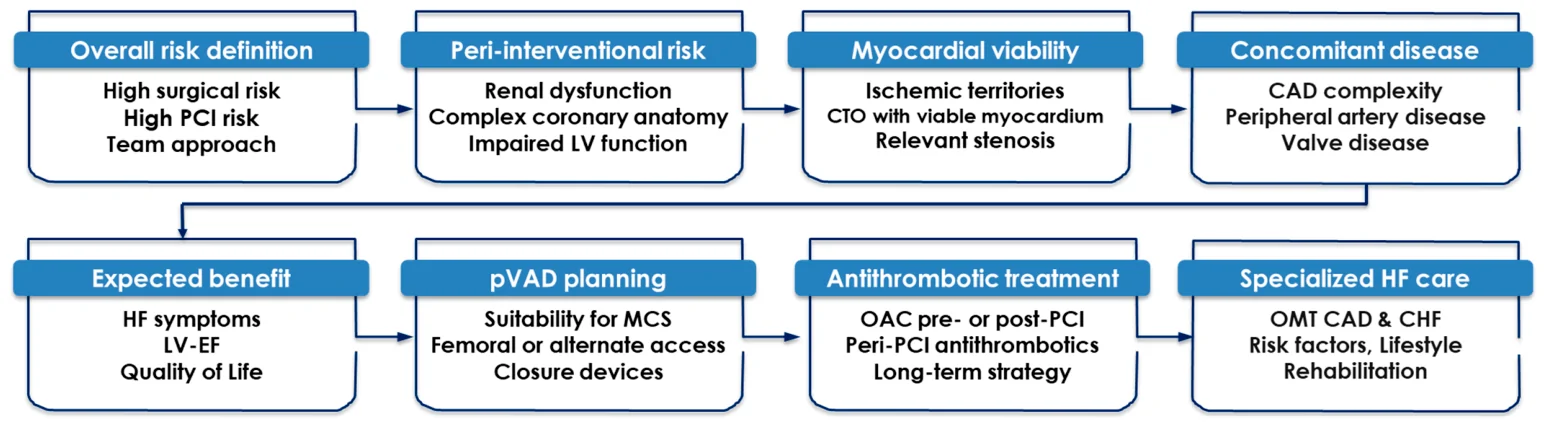
Stepwise approach to the *protected high-risk PCI* indication for mAFP in patients with HF and extensive, symptomatic coronary artery disease. CAD = coronary artery disease; CHF = congestive heart failure; CTO = chronic total occlusion; HF = heart failure; LV = left ventricular; LV-EF = LV ejection fraction; MCS = mechanical circulatory support; OAC = oral anticoagulation; OMT = optimal medical therapy; PCI = percutaneous coronary intervention; pVAD = percutaneous ventricular assist device.

As long as the use of protected high-risk PCI remains based on individual decision-making, a structured, stepwise approach encompassing procedural planning and execution is recommended [18], as outlined in Figure 2.

**Figure 3:**
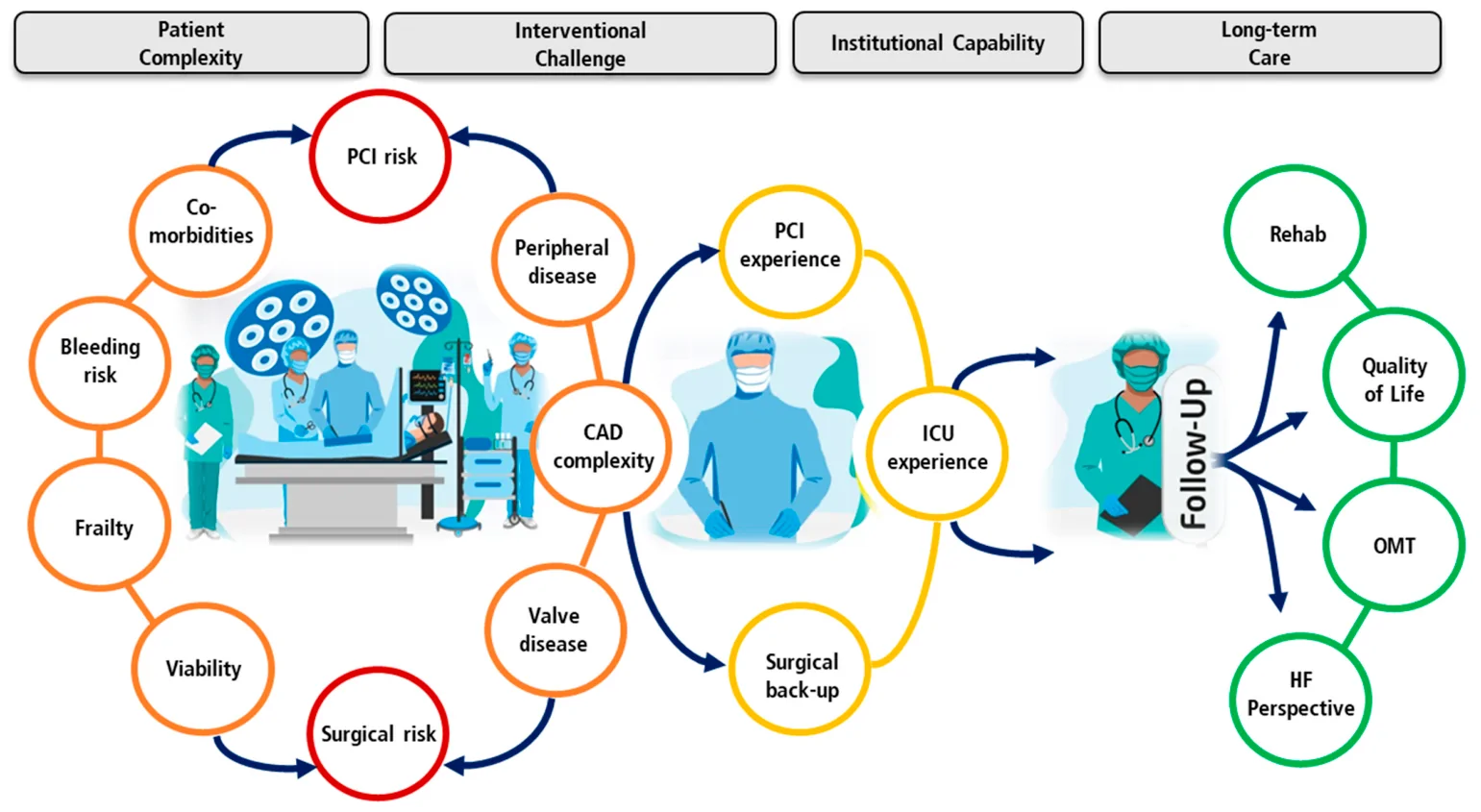
For the *elective coronary* indication for mAFP, so-called protected high-risk PCI, careful assessment of PCI and surgical risk revealing a surgical risk exceeding the risk of PCI, if haemodynamic collapse can be prevented by mAFP placement, is required. There are certain restrictions that need to be carefully evaluated to assess the patient complexity owing to comorbidities, bleeding risk, frailty, and viable and hence potentially salvageable ischemic myocardium. The interventional challenge is not only restricted to coronary artery disease (CAD) complexity, but is potentially aggravated by peripheral and/or valve disease complicating device placement. Pairing PCI experience in the cath lab intended to overcome the interventional challenges in complex CAD, comfort to implant mAFP, experience in caring for patients on mAFP during a stay on an intensive care unit (ICU)—if required—and the potential cardiac or vascular surgery back-up is an institutional mandatory framework. If all of these play together, the patient might benefit from ischemia relief and should receive formalised and structured follow-up guidance. OMT = optimal medical therapy.

Although positive trial data are clearly needed to support the widespread use of expensive MCS devices for indications such as protected high-risk PCI, institutions offering these procedures should also optimise their local infrastructure and processes at the institutional, patient, and procedural levels. The key factors to consider when establishing such a programme are illustrated in Figure 3.

### 2.2. Acute Myocardial Infarction-Related Cardiogenic Shock (AMI-CS)

Mortality among patients with cardiogenic shock (CS) remains high, at approximately 50% in RCTs conducted over recent decades, despite advances in PCI [6,31,32,33,34,35]. Although various MCS devices can improve haemodynamic stability, neither veno-arterial extracorporeal membrane oxygenation (VA-ECMO) nor the intra-aortic balloon pump (IABP) has demonstrated a significant reduction in mortality in randomised trials [35,36]. Given that the vast majority of patients enrolled in IABP-SHOCK II received IABP support only after PCI had been completed, it remains uncertain whether pre-PCI insertion would have resulted in a different outcomes. Two relevant RCTs have investigated using VA-ECMO in this setting, although the smaller trial did not include patients who had undergone resuscitation [32,37]. Neither trial demonstrated a mortality benefit associated with the routine use of VA-ECMO in AMI-CS. Accordingly, current American guidelines do not recommend its routine use in this setting [1].

Retrospective studies of mAFP use have suggested a potential benefit, particularly when the device was implanted before PCI and used in patients at higher risk of mortality [10,38,39,40,41,42,43]. In 2024, the landmark DanGer-Shock trial demonstrated, for the first time, a significant reduction in mortality in an adequately powered RCT investigating the early use of an mAFP in patients with AMI-CS [2]. The benefit was demonstrated for the primary endpoint at 180 days and was sustained during long-term follow-up extending to 10 years [44]. More detailed analysis of the trial data suggested that the benefit was more pronounced in patients younger than 75 years [45]. In addition, a subgroup analysis of patients with multivessel disease suggested a benefit associated with early complete revascularisation in the DanGer-Shock population [46]. These findings warrant consideration in the context of CULPRIT-SHOCK, which did not demonstrate a benefit of complete revascularisation over culprit-lesion-only revascularisation in patients with AMI-CS [34]. Subsequent analyses of CULPRIT-SHOCK, however, suggested that the lack of benefit associated with the multivessel strategy was partly attributable to a high proportion of patients in whom revascularisation remained incomplete. Patients in whom complete revascularisation was achieved had better outcomes [47]. These findings are also supported by data from the Italian national Impella registry, IMP-IT, which demonstrated an association between complete revascularisation and reduced mortality in patients with AMI-CS supported by an mAFP [43]. When interpreting the positive results of DanGer-Shock and their implications for future AMI-CS management, it is important to recognise that the trial selected patients with predominantly LV failure, in whom loss of myocardial contractility resulted in LV overload. As LV-unloading devices, mAFPs require sufficient LV filling to function effectively. Patients with overt right ventricular (RV) failure were therefore excluded from DanGer-Shock. In addition, patients who remained comatose following cardiac arrest were excluded to avoid substantial confounding of the primary endpoint by hypoxic–ischaemic brain injury that may have occurred before trial enrolment [48,49].

Several subgroup and secondary analyses of DanGer-Shock have provided more detailed insights into the factors influencing outcomes. Although mAFP treatment reduced mortality in patients with AMI-CS, the apparent benefit diminished as the interval between symptom onset and device implantation increased. The lowest mortality was observed when implantation occurred within three hours of symptom onset [50]. Although the initial results suggested a smaller treatment effect in women, subsequent adjustment for potential confounders, including age and delays in presentation, indicated that the benefit associated with mAFP treatment was comparable in women and men [51].

**Figure 4:**
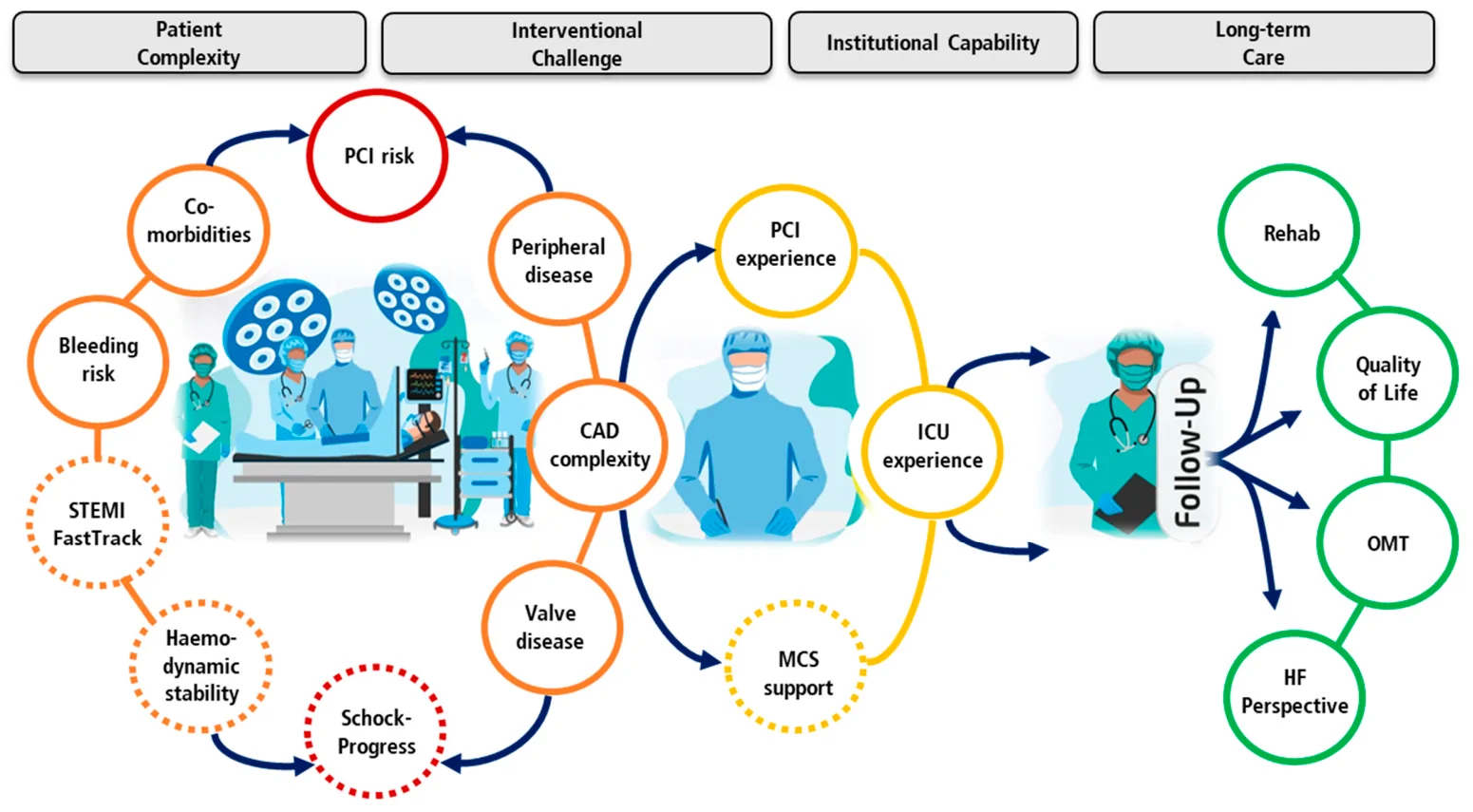
For the *emergent* coronary indication for mAFP, there are some modifications compared to the elective coronary set-up as outlined in Figure 2. Most importantly, the risk of a PCI procedure with or without mAFP is now balanced to shock progression without LV unloading. In general, haemodynamic instability and the inability to prepare for the procedure due to an emergent STEMI-fast track admission add complexity to the procedure as many crucial steps have to either be pre-set by a standardised shock protocol (preferable) or happen based on individual and impulsive improvisation (to be kept to a minimum). For the interventional and institutional setting, surgical back-up is not the driving factor anymore, but experience with short- as well as long-term use and handling of MCS devices becomes much more important.

Despite the mortality reduction observed in appropriately selected patients, MCS devices are associated with an increased risk of bleeding. Patients treated with an mAFP experienced more bleeding events than patients with AMI-CS who did not receive device support, and the risk varied according to the complexity of the MCS strategy. The reported risk of bleeding increased approximately 5-fold with mAFP implantation, 8-fold with VA-ECMO, and 27-fold with combined mAFP and VA-ECMO. Nevertheless, bleeding events did not appear to negate the mortality benefit associated with mAFP treatment in patients with AMI-CS [52]. Institutions providing primary PCI for patients with AMI-CS should ensure that their catheterisation laboratories are organised to permit the rapid insertion and activation of an mAFP whenever indicated by patient characteristics and procedural circumstances. The key organisational and procedural factors to consider when establishing such a programme are illustrated in Figure 4.

One of the major unresolved challenges in AMI-CS management is the prospective identification of patients who are likely to require and benefit from mAFP implantation. Analyses of DanGer-Shock identified several patient characteristics associated with a greater likelihood of recovery: younger age (odds ratio [OR], 0.65 per 10-year increase), higher LVEF (OR, 1.88 per 10-percentage-point increase), lower admission lactate levels (OR, 0.39 per doubling of lactate concentration), and lower heart rate (OR, 0.82 per 10 beats/min increase) [53]. An alternative approach is to identify the underlying CS phenotype. Although the cardiometabolic phenotype is associated with the highest mortality, it may represent an advanced stage of shock progression that is already difficult to reverse. By contrast, the non-congested phenotype may represent a more promising treatment target, as mAFP implantation was associated with a significant reduction in observed mortality in this subgroup [54,55].

In light of the DanGer-Shock results, mAFPs have become an important component of the therapeutic armamentarium for AMI-CS. Accordingly, the 2025 American guidelines on acute coronary syndromes and the 2026 European guidelines on heart failure recommend considering mAFP support in selected patients with AMI-CS [1,56].

### 2.3. Heart Failure-Related Cardiogenic Shock (HF-CS)

In contrast to AMI-CS, which often has a sudden and clearly identifiable onset and may result from a treatable and reversible cause, HF-CS is more heterogeneous in its underlying aetiology and frequently develops as a consequence of recurrent decompensation. Hence, the opportunity for timely intervention and reversal of the underlying process is often more limited. Previous RCTs investigating other MCS devices in this setting have not demonstrated a clear benefit. A trial evaluating IABP in patients with HF-CS, ALTSHOCK-2, was designed to enrol 200 patients. Patients were excluded if shock was secondary to cardiac or non-cardiac surgery, a recent myocardial infarction within the preceding month, suspected or confirmed myocarditis, pulmonary embolism, or chronic end-stage organ dysfunction that would preclude heart transplantation or a durable LV assist device (LVAD) as destination therapy. After approximately half of the planned population had been enrolled, an interim analysis suggested that the experimental treatment was unlikely to provide meaningful benefit. The trial was therefore terminated prematurely, and the results were published on the basis of 101 patients. The enrolled population exhibited considerable variation in shock severity: only 48% had an admission lactate concentration above 2 mmol/L, 24% had oliguria, 57% were classified as Society for Cardiovascular Angiography and Interventions (SCAI) stage C, 16% as stage D, and 28% as stage B. One-third of the patients did not receive inotropic support. The primary endpoint, defined as 60-day survival or successful bridging to advanced heart replacement therapy, did not differ significantly between patients treated with IABP and those receiving the comparator strategy [57].

Although ALTSHOCK-2 investigated IABP rather than an mAFP, it illustrates several fundamental challenges in conducting clinical trials of MCS in HF-CS. First, the patient population is highly heterogeneous with respect to shock duration and severity, underlying disease, progressive chronic HF, reversibility, and the degree of circulatory support required. Second, recruitment may be challenging, particularly in patients with less severe or less acute presentations, while the potential complications associated with large-bore devices, including bleeding, remain relevant. Taken together, these limitations suggest that conducting a large, adequately powered RCT in this patient population may be difficult in the near future.

From a mechanistic perspective, mAFPs can increase coronary flow reserve in patients with severe non-ischaemic dilated cardiomyopathy [17]. Their use may therefore improve the haemodynamic tolerance of procedures that would otherwise further compromise cardiac function. However, this approach does not specifically treat the underlying myocardial disease. In observational studies, patients with HF-CS treated with an mAFP have exhibited lower mortality than patients with AMI-CS, most likely reflecting differences in shock severity and the underlying clinical characteristics of the two populations rather than a differential treatment effect [58].

### 2.4. Terminal Heart Failure—Bridge to Durable Assist Implantation or Heart Transplantation

Temporary MCS is strongly recommended for patients with advanced HF who deteriorate while awaiting durable LVAD implantation or heart transplantation. In these patients, optimisation of end-organ function before definitive treatment should be considered. For patients being evaluated for durable LVAD implantation who are at risk for RV failure, temporary RV support may also be considered to facilitate perioperative haemodynamic stabilisation [59].

Implantation of a durable LVAD in patients presenting with CS or severely impaired haemodynamics, particularly those classified as INTERMACS 1 or 2, is associated with substantially worse postoperative outcomes [60,61,62]. By contrast, patients who reach INTERMACS profile 3 or 4 before durable LVAD implantation have considerably better outcomes, with reported one-year survival approaching 80%, compared with approximately 50% among patients implanted at INTERMACS profiles 1 or 2. Post-implantation survival is influenced not only by the patient’s clinical status before surgery but also by the type of temporary MCS used as a bridge to durable LVAD implantation [61,62].

**Figure 5:**
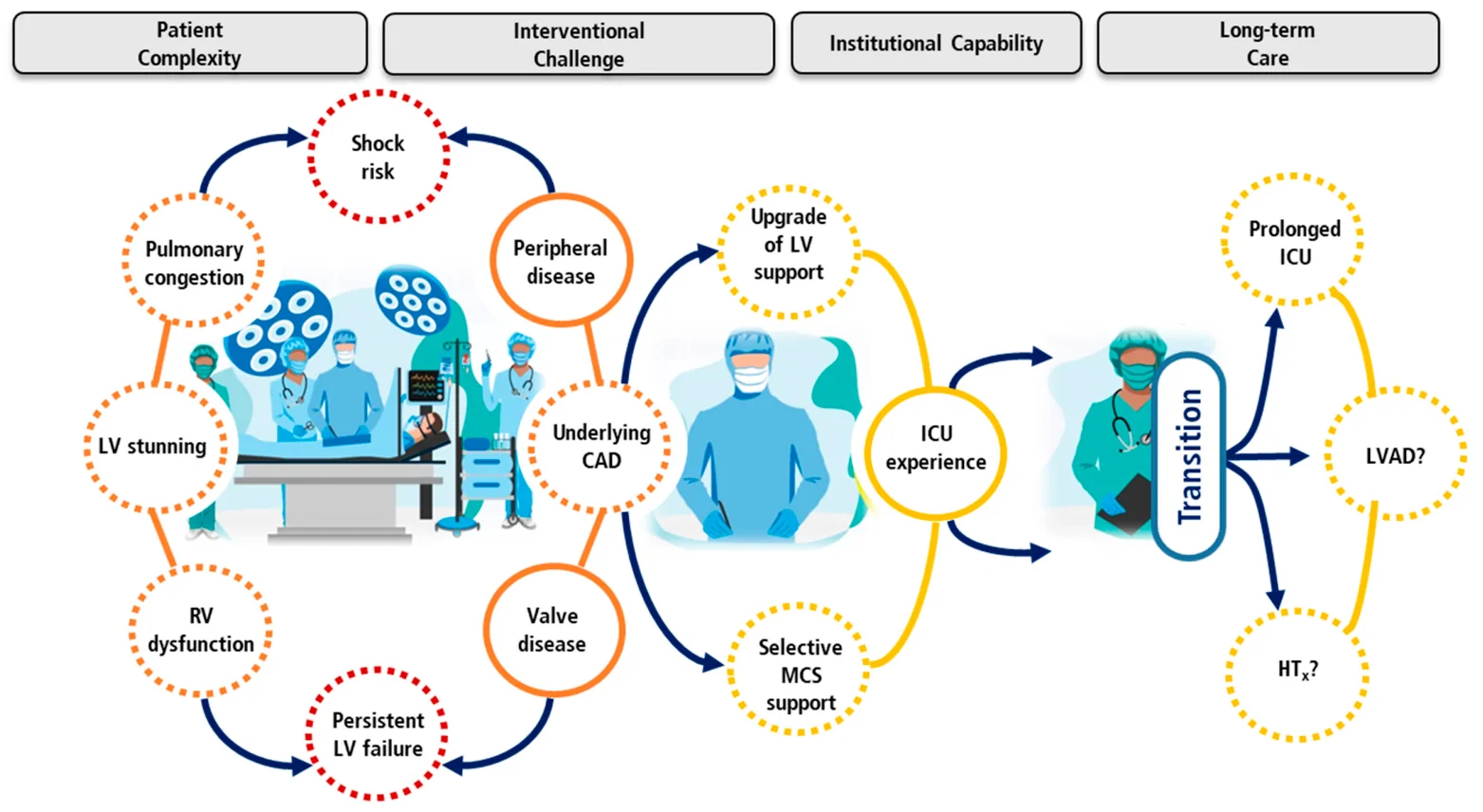
For the *preconditioning* indication for mAFP, there are some modifications compared to the previous set-ups. Most importantly, the risk of persistent LV failure or necessity of higher LV support demand or potential passive RV unloading is dominating the approach. It is also a test, whether the support provided by certain devices such as Impella 5.5 mimicking the higher support of a durable LVAD can accommodate the patient’s demand or whether a transplant is required. The failure to determine the presence of a specific treatment to the cause of cardiogenic shock requires ICU staff to be competent and confident to handle patients on multiple interacting MCS devices. The usual follow-up is now substituted by intermediate-to-long-term ICU care acting to bridge the patient to transplantation or implantation of a durable LVAD.

These observations have contributed to the concept of preconditioning with mAFP support before heart transplantation. A key advantage of the strong haemodynamic support provided by an Impella 5.5 inserted through an axillary or subclavian approach is that patients can be mobilised before transplantation, potentially limiting peripheral skeletal muscle wasting and physical deconditioning. In a retrospective cohort study of 65 patients who underwent preconditioning with an Impella 5.5, greater walking distances during device support in the intensive care unit (ICU) were associated with shorter hospital stays and earlier weaning from mechanical ventilation after transplantation. The optimal recovery period appeared to be within the first two to three weeks of support, with diminishing additional recovery thereafter. This suggests that much of the functional improvement may occur during the initial phase of preconditioning [63]. Other important aspects of preconditioning include adequate decongestion while preventing cardiorenal decompensation [64]. The institutional infrastructure and organisational considerations relevant to establishing a preconditioning programme are illustrated in Figure 5.

### 2.5. LV Unloading in vaECMO Patients

Different MCS devices have distinct effects on haemodynamic parameters, particularly LV end-diastolic pressure (LVEDP) and LV loading conditions. Whereas IABP has a relatively neutral effect on LV loading and LVEDP, VA-ECMO increases LV afterload and may thereby exacerbate LV overload [36]. In contrast, mAFPs actively unload the LV. Experimental studies have demonstrated that increasing VA-ECMO blood flow in models of CS can adversely affect LV function [65,66,67]. Recognition of these effects has led to interventional strategies in which mAFPs are used to actively unload the LV in patients primarily supported with VA-ECMO for CS [68,69,70]. Although this approach is physiologically appealing, the additional risks of bleeding, haemolysis, and renal failure must be considered [52,69]. Early propensity score-matched analyses suggested that patients receiving combined VA-ECMO and Impella support, commonly referred to as ECMELLA, had lower in-hospital mortality and higher rates of successful bridging to myocardial recovery or definitive advanced heart failure therapy than patients receiving VA-ECMO alone. However, combined support was also associated with a greater need for renal replacement therapy and higher incidence of haemolysis. The findings regarding major bleeding were inconsistent across analyses [69,71].

Prospective evidence remains limited. The EARLY-UNLOAD trial, which investigated transseptal cannulation for left atrial unloading in patients receiving VA-ECMO, did not demonstrate a mortality benefit from early unloading. Interpretation of the results was complicated by a high rate of rescue transseptal cannulation in the control group, which was associated with worse outcomes [72]. By contrast, a larger, contemporary, propensity score-matched analysis comparing 510 patients receiving VA-ECMO with or without additional mAFP support found that LV unloading was associated with lower mortality. However, complications were more frequent in patients receiving combined support, including severe bleeding, access site-related limb ischaemia, abdominal compartment syndrome, and the need for renal replacement therapy [73].

**Figure 6:**
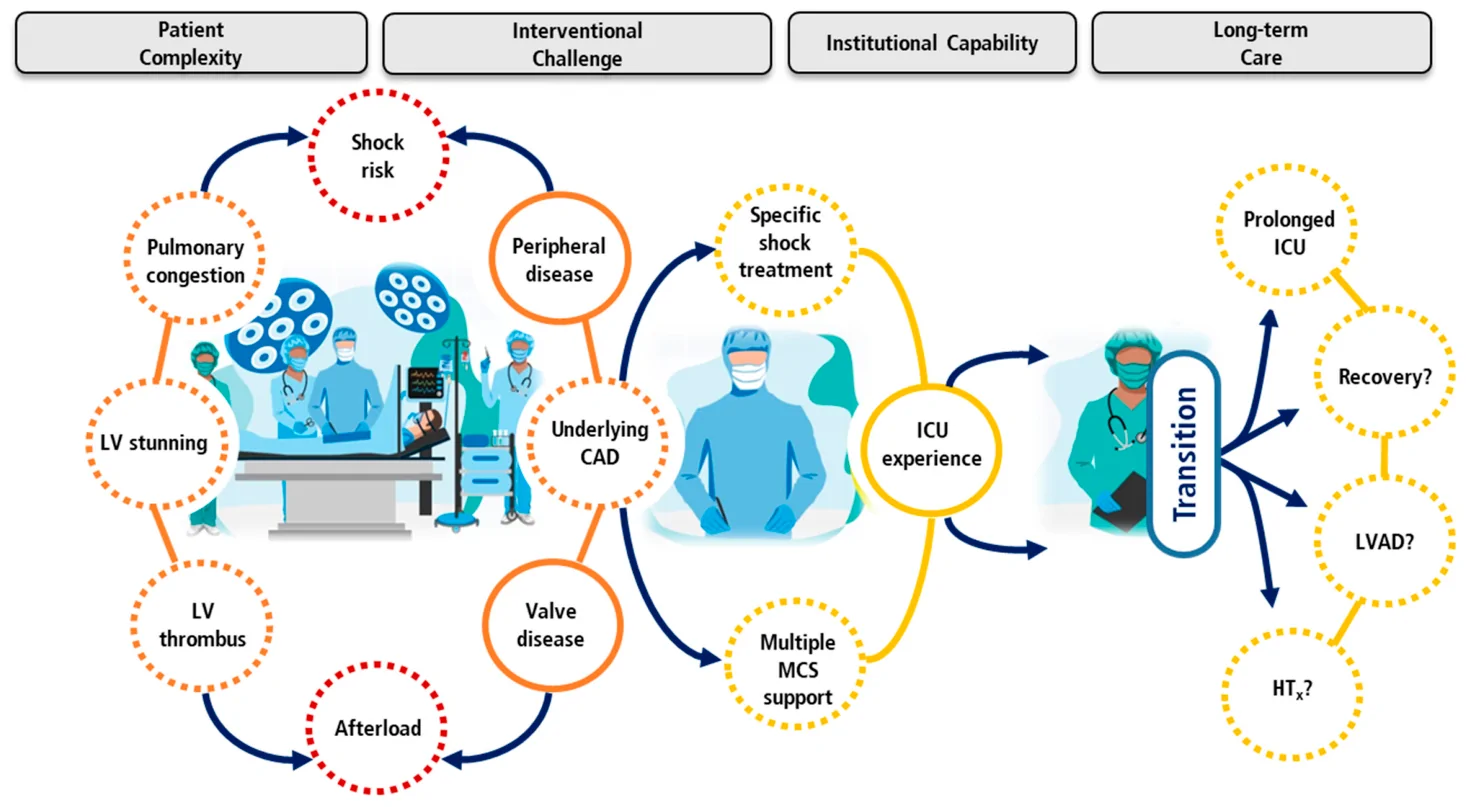
For the *ECMELLA* indication for mAFP, there are some modifications compared to the other coronary set-ups. Most importantly, the risk of LV distension caused by retrograde arterial blood flow against a compromised LV increases the risk for further LV compromise, potentially resulting in intraventricular thrombus formation and/or severe pulmonary congestion. Acute success is determined by the presence of a specific treatment for the cause of cardiogenic shock and ICU staff need to be competent and confident to handle patients on multiple interacting MCS devices. The usual follow-up is now substituted by intermediate-to-long-term ICU care potentially aiming for recovery or bridging the patient to transplantation or a durable LVAD.

Before ECMELLA is adopted more widely, its efficacy and safety need to be established in adequately powered prospective trials. The UNLOAD-ECMO trial (NCT05577195) was designed to randomise 198 patients with CS to VA-ECMO with additional mAFP-mediated LV unloading or VA-ECMO alone [74]. Until this trial is completed, the use of an mAFP for LV venting in patients receiving VA-ECMO should be considered on an individual basis and supported by an appropriate institutional infrastructure, as illustrated in Figure 6.

### 2.6. RV Unloading and Bypass in Right Ventricular Failure

Acute isolated RV failure is a serious clinical complication that may occur during LV unloading or in the course of CS more generally. It can arise as a primary consequence of RV myocardial dysfunction, for example due to ischaemia or arrhythmia, or develop secondarily in response to an acute increase in RV afterload, as occurs in pulmonary embolism and other conditions associated with elevated pulmonary vascular resistance.

Right-sided heart failure is frequently under-recognised and may be misinterpreted as global cardiac or isolated LV pump failure [75]. The RV has limited compensatory capacity and a relatively low contractile reserve. Treatment strategies include pharmacological interventions, such as inotropic support, reduction in RV afterload, and optimisation of volume status. In selected patients, MCS may also be required. Temporary RV support can be provided by transvenously implanted mAFPs. The Impella RP is advanced from the femoral vein through the inferior vena cava and right atrium, across the tricuspid valve and RV, and into the pulmonary artery, where the outflow segment is positioned. The considerable intravascular distance traversed by the device may increase the risk of migration and suboptimal positioning. The Impella RP can improve survival and clinical outcomes in selected patients with RV dysfunction following LVAD implantation, postcardiotomy syndrome, or myocardial infarction [76,77].

The Impella RP Flex represents a further development of this technology. Inserted via the internal jugular vein, it offers improved positional stability and facilitates near-complete patient mobilisation [78]. Unlike ECMO-based support systems, mAFPs do not provide respiratory support or extracorporeal gas exchange. Their role is therefore limited to haemodynamic support of the circulation.

### 2.7. LV Unloading in Postcardiotomy Shock or Low Cardiac Output Syndrome After Cardiac Surgery

The incidence of low cardiac output syndrome (LCOS) following cardiac surgery has been reported to be as high as 27%, although rates vary according to procedural complexity, pre-existing cardiac function, and the burden of comorbidities. Patients undergoing combined mitral valve surgery and coronary artery bypass grafting appear to be at particularly high risk for developing LCOS [79].

The 2025 joint guidelines from the European Association for Cardio-Thoracic Surgery, the Society of Thoracic Surgeons, and the American Association for Thoracic Surgery provide a comprehensive consensus framework for the use of temporary MCS in adult cardiac surgical practice. They recommend the use of a full-flow microaxial pump, such as the Impella 5.5, in patients with post-procedural CS. Similarly, patients who cannot be weaned from cardiopulmonary bypass after cardiac surgery and who are suitable candidates for MCS should undergo immediate escalation to mechanical support [59]. From an institutional perspective, implementation of this strategy does not necessarily require substantial changes to the standard perioperative care pathway. The decision to initiate MCS is made in the operating theatre, where the patient is already receiving cardiopulmonary bypass, and postoperative intensive care takes place in an environment generally equipped to manage patients either on ECMO or a durable LVAD. Nevertheless, appropriate protocols, device availability, and coordination between cardiac surgeons, cardiologists, anaesthesiologists, and intensive care teams remain essential.

### 2.8. Protected Cardiac Surgery

The principle of prevention rather than rescue has become an established rationale for protected high-risk PCI, particularly in patients who are not suitable candidates for conventional surgical revascularisation [18]. Building on the approaches described above, including preconditioning patients with severe LV dysfunctional before cardiac surgery and experience gained in the management of postcardiotomy shock, the concept of protected cardiac surgery extends the use of mAFPs to planned haemodynamic support during cardiac surgical procedures. The underlying rationale is to prevent postcardiotomy cardiac failure by providing circulatory support before severe haemodynamic deterioration occurs.

Recent consensus statements from leading professional societies in the United States and Europe distinguish three approaches to protected cardiac surgery: preoperative support as a bridge to surgery, planned intraoperative support, and rescue intraoperative support. In the latter scenario, MCS is initiated without delay, with immediate transition from cardiopulmonary bypass to MCS when required [80,81].

Following initial single-centre experiences and studies investigating this approach [82], a prospective multicentre registry trial in the United States and Europe, the IMpella-Protected cArdiaC Surgery Trial (IMPACT; NCT05529654), was conducted to evaluate the safety and feasibility of this strategy. This prospective, multicentre, single-arm study assessed the initiation of transvalvular LV unloading before separation from cardiopulmonary bypass. A total of 100 patients received an Impella 5.5. The primary effectiveness endpoint, defined as in-hospital postcardiotomy cardiac failure, occurred in 6% of patients. The primary safety endpoint, a composite of all-cause mortality, stroke, and new-onset renal replacement therapy within 90 days after surgery, occurred in 12%. Mortality was 2% at 30 days and 4% at 90 days. Overall, Impella 5.5-protected cardiac surgery appeared to be feasible, with an acceptable safety profile in selected patients with severe LV dysfunction [20]. However, the single-arm design limits the extent to which these findings can establish comparative efficacy relative to conventional surgical management.

### 2.9. LV Unloading Prior to PCI in Non-Shock Acute Myocardial Infarction (AMI) Without Cardiogenic Shock

As increasing attention is directed towards preventing haemodynamic deterioration rather than treating established circulatory collapse, interest has shifted towards strategies aimed at preventing the development of heart failure and severe LV dysfunction following extensive acute myocardial infarction (AMI). Various pharmacological and interventional approaches have been investigated to mitigate reperfusion injury, limit myocardial necrosis and thereby reduce the risk of subsequent severe LV dysfunction [83,84]. Following the widespread implementation of AMI fast-track programmes, the contribution of ischaemic injury to final infarct size has decreased. Nevertheless, infarct size remains substantial, particularly in patients presenting with large anterior AMIs, in whom the infarct may involve approximately 30% of LV mass [84,85]. Numerous pharmacologic and interventional strategies have been investigated to further limit infarct size, but few have demonstrated convincing clinical benefit [84]. Among these approaches, intracoronary administration of super-saturated oxygen has demonstrated a reduction in infarct size and microvascular obstruction [85,86,87]. Both parameters are relevant surrogate markers associated with mortality and hospitalisation for HF [88,89].

In animal models of AMI, mAFP implantation followed by LV unloading prior to revascularisation substantially reduced infarct size [90,91]. In an initial clinical pilot trial investigating the feasibility of this approach in humans, patients with particularly large AMIs appeared to benefit from LV unloading with an mAFP for 30 min before reperfusion of the occluded coronary artery [92]. The hypothesis that LV unloading for 30 min before revascularisation could reduce infarct size and potentially prevent subsequent HF hospitalisations was investigated in the adequately powered STEMI-DTU trial. A total of 527 patients with acute anterior AMI without shock were enrolled and randomised to either standard care with immediate revascularisation or LV unloading for 30 min followed by deferred revascularisation. The trial did not demonstrate a significant reduction in infarct size with the unloading strategy: infarct size was 30.8% of LV mass in the mAFP group and 31.9% in the control group (*p* = 0.50). Furthermore, the strategy exposed patients to potential harm associated with large-bore vascular access, including bleeding and vascular complications [93].

Thus, despite encouraging preclinical evidence suggesting that LV unloading before revascularisation may reduce infarct size in patients with AMI without cardiogenic shock, the adequately powered RCT failed to demonstrate a significant benefit for this surrogate endpoint. The procedure was also associated with additional procedural risks, particularly bleeding complications. Accordingly, routine LV unloading before revascularisation cannot currently be recommended for patients with AMI without cardiogenic shock outside established indications for MCS, such as selected cases of cardiogenic shock or protected high-risk PCI.

### 2.10. Protected Ablation of Ventricular Tachycardia

The concept of mAFP-supported high-risk PCI is based on the ability of these devices to prevent haemodynamic compromise during complex and prolonged revascularisation procedures in patients with severely impaired LV function. In these patients, procedural ischaemia may critically compromise myocardial oxygen supply and potentially trigger ventricular arrhythmias [18,19]. Early animal experiments provided a pathophysiological rationale for LV unloading in this context. In pigs with experimentally induced AMI, reduced LVEF, and elevated LVEDP, mAFP-mediated LV unloading reduced LVEDP, accompanied by proportional decreases in mean left atrial pressure and maximum left atrial volume. These changes in turn reduced left atrial stretch and were associated with lower atrial arrhythmogenicity [94]. Building on these pathophysiological considerations and early experiences with MCS as a rescue strategy, the concept of MCS-supported ventricular tachycardia (VT) ablation has evolved. During these procedures, haemodynamic support aims to maintain adequate end-organ perfusion during sustained episodes of VT, thereby allowing detailed mapping of the re-entry circuit [95].

**Figure 7:**
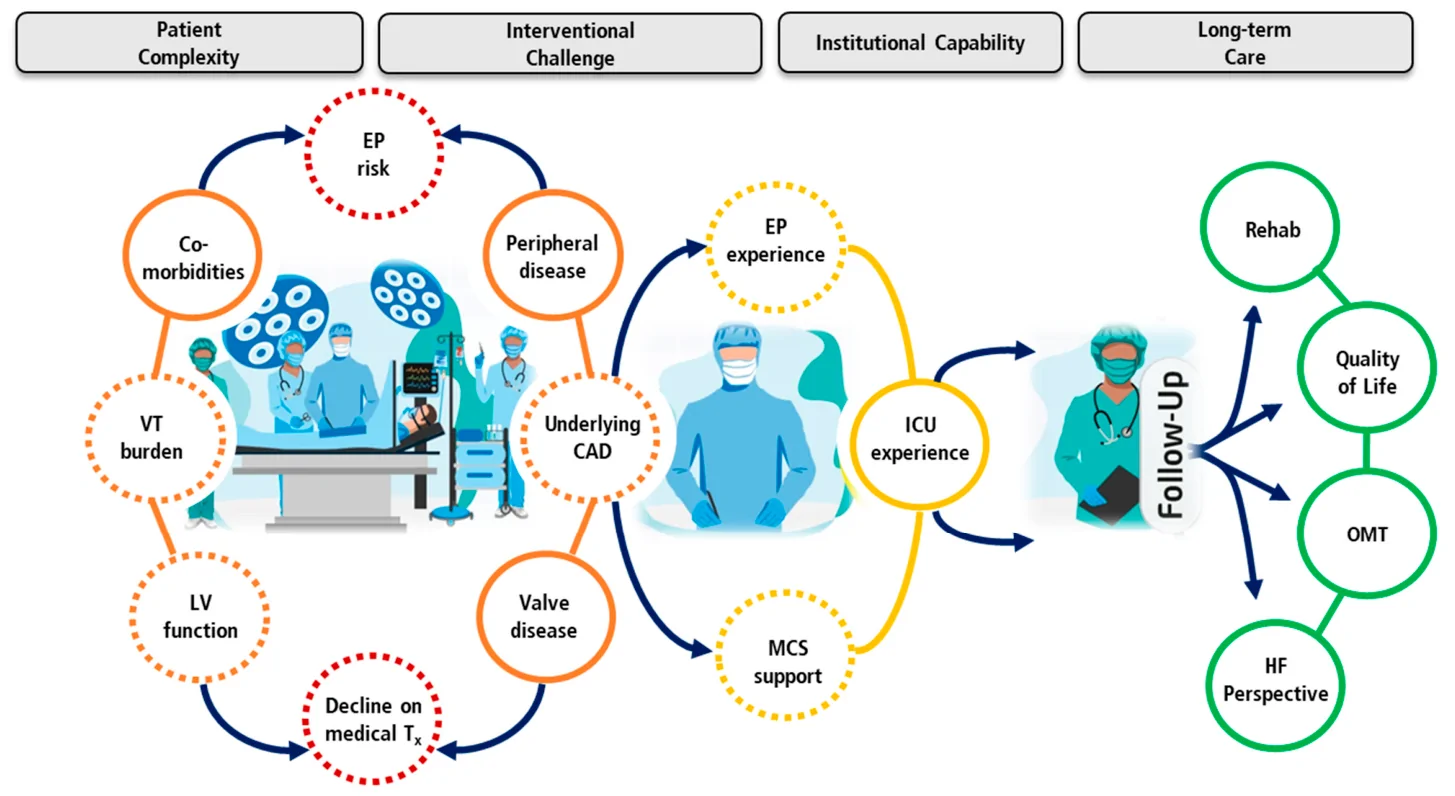
For the *non-coronary* indication for mAFP, there are some modifications compared to the elective coronary set-up as outlined in Figure 2. Most importantly, now the risk of ventricular tachycardia (VT) ablation procedure with or without mAFP is balanced to decline on medical therapy without VT ablation. In general, the VT burden as well as the underlying LV function influence stability. A co-existent coronary artery disease might further impact the perceived haemodynamic stability during VT required for ablation. For the interventional and institutional setting, electrophysiology (EP) capabilities to resolve the VT burden and overall institutional experience with short- as well as long-term use and handling of MCS devices becomes much more important.

Potential benefits of MCS during VT ablation include the maintenance of vital organ perfusion, reductions in intracardiac filling pressures and LV volumes, lower myocardial wall stress and oxygen consumption, and improved coronary perfusion during prolonged VT induction and mapping. Mechanical support may permit longer mapping periods, facilitate more comprehensive characterisation of the arrhythmogenic substrate, and potentially increase the likelihood of successful VT termination. It may also allow patients with more advanced disease to undergo ablation. However, this approach has potential disadvantages, including increased risks of vascular, bleeding, or thromboembolic complications, as well as greater procedural complexity and cost. Acute haemodynamic decompensation during VT ablation is associated with increased mortality, emphasising the importance of prospectively identifying patients at risk [96]. Initial observational studies of MCS-supported, or “protected”, VT ablation have shown that patients receiving such support tend to have more advanced disease. Despite similar rates of acute procedural success, VT recurrence, and mortality, MCS use has been associated with higher complication rates and longer fluoroscopy and procedure time [21,97]. Based on the available observational evidence, protected VT ablation may be considered in patients in whom an unsupported procedure would be expected to cause intolerable haemodynamic compromise. This remains an individualised decision and should follow a structured approach similar to the one suggested for protected high-risk PCI [18]. The key factors to consider when establishing a programme are illustrated in Figure 7.

An important challenge when considering elective protected VT ablation in patients with severely impaired LV function is that the arrhythmia is often a consequence of the underlying myocardial disease. Unlike revascularisation during protected PCI, VT ablation does not directly treat the underlying myocardial dysfunction and may temporarily exacerbate LV impairment. Consequently, even after successful ablation, a patient may be unable to tolerate withdrawal of MCS. Careful patient selection is therefore essential to minimise the risk of prolonged dependence on mechanical support. Preprocedural assessment should include consideration of potential bailout strategies, including whether the patient would be a suitable candidate for durable LVAD implantation or prolonged temporary MCS support on an intensive care unit.

### 2.11. Protected Non-Cardiac Surgery

**Figure 8:**
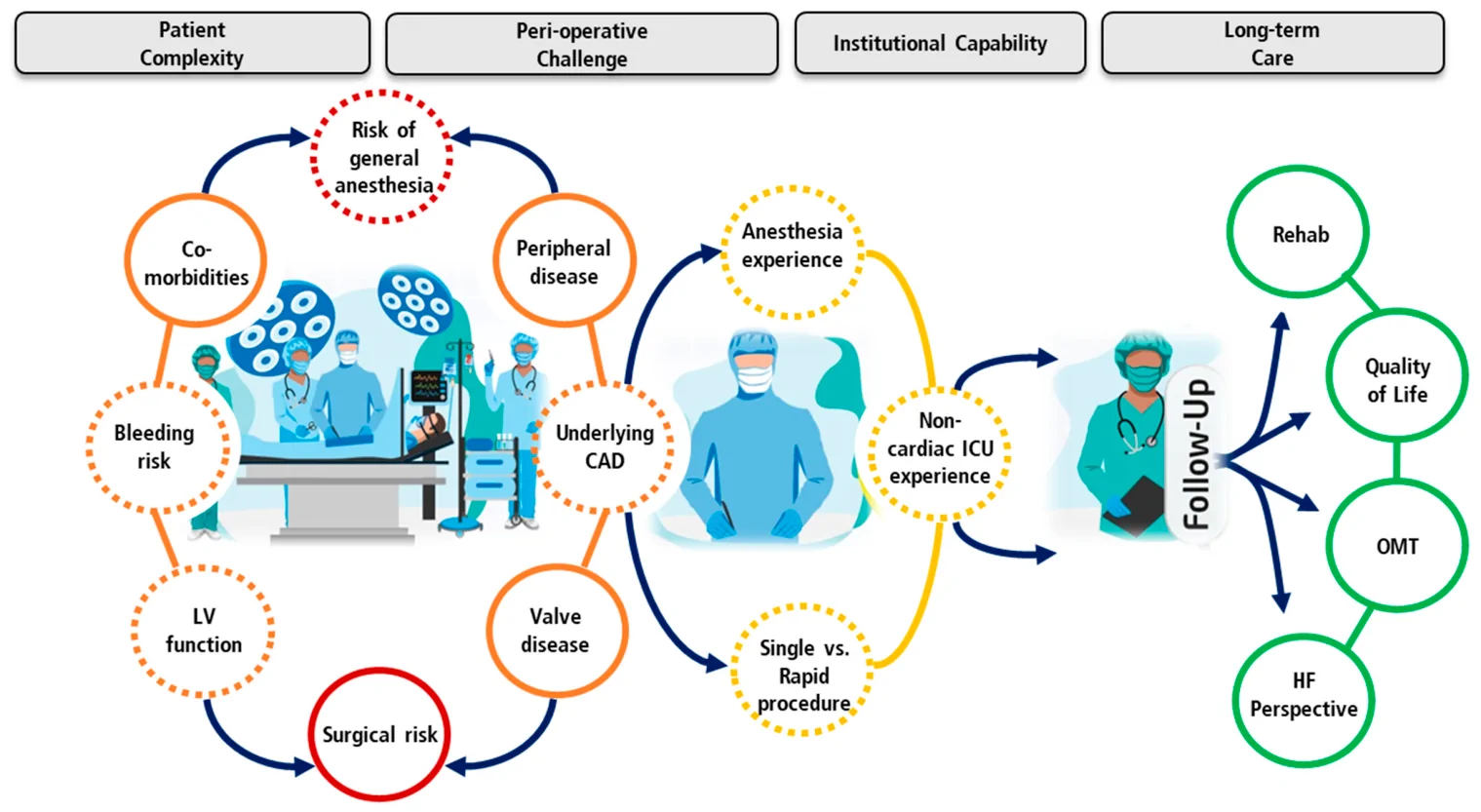
For the *non-cardiac indication* for mAFP in surgery in patients with severe HF/LV dysfunction, there are some modifications compared to the elective coronary set-up as outlined in Figure 2. Most importantly, now the risk of general anaesthesia is added to the surgical risk in a patient with low tolerance to volume shift and/or low functional haemodynamic reserve. In general, the bleeding risk as well as the underlying LV function influence stability. A co-existent coronary artery disease might further affect the perceived haemodynamic stability during general anaesthesia for the non-cardiac surgery. For the perioperative and institutional setting, overall institutional experience (additive cardio-anaesthetist and/or cardiologist) with advanced haemodynamics as well as short-term use and handling of MCS devices is paramount in addition to procedural planning that tries to avoid multiple or repetitive surgical procedures and fix the surgical problem in one protected procedure. Furthermore, definitive placement on intensive care has to be pre-organised including potential placement of the patient in an environment convenient for MCS devices.

Advances in the management of acute cardiogenic shock and the revascularisation of patients with severe HF have enabled an increasing number of patients to survive acute illness and continue treatment in the outpatient setting. With improvements in pharmacological therapy, some patients with severe HF can maintain a relatively good quality of life despite substantial underlying cardiac dysfunction. Consequently, these patients may require non-cardiac surgery for indications unrelated to their cardiac disease, as do individuals without HF. Nevertheless, patients with severe chronic HF have a substantially increased risk of perioperative mortality when undergoing a range of non-cardiac surgical procedures [98]. By analogy with protected high-risk PCI and high-risk cardiac surgery, it is therefore conceivable that temporary MCS during non-cardiac surgery could help to stabilise haemodynamics and mitigate some of the risks associated with LV dysfunction. Several investigators have recently reported the use of mAFPs during different types of non-cardiac surgeries in patients with chronic HF or acute haemodynamic compromise [22,23,99]. The key institutional and procedural factors to consider when implementing this approach are illustrated in Figure 8.

The reported experiences encompass different clinical scenarios. In some cases, non-cardiac surgery was performed in patients already receiving MCS for an acute HF or CS [99,100]. In others, mAFPs were implemented electively to provide planned haemodynamic support during surgery in patients with severe HF [23,99]. Procedures described in the literature include, but are not limited to, thyroidectomy, cholecystectomy, and bariatric surgery.

Despite the absence of RCTs specifically evaluating MCS during non-cardiac surgery in patients with severe HF or LV dysfunction, individualised decision-making may consider whether perioperative haemodynamic stabilisation with an mAFP could facilitate surgery and reduce the risk of deterioration in patients with limited cardiac reserve.

On the basis of the available observational evidence, protected non-cardiac surgery may be considered when surgery or anaesthesia without MCS is expected to cause haemodynamic compromise in patients with severe HF or LV dysfunction. As with protected high-risk PCI, the decision should be individualised and based on a structured assessment of the anticipated benefits, procedural risks, and available alternatives [18].

**Figure 9:**
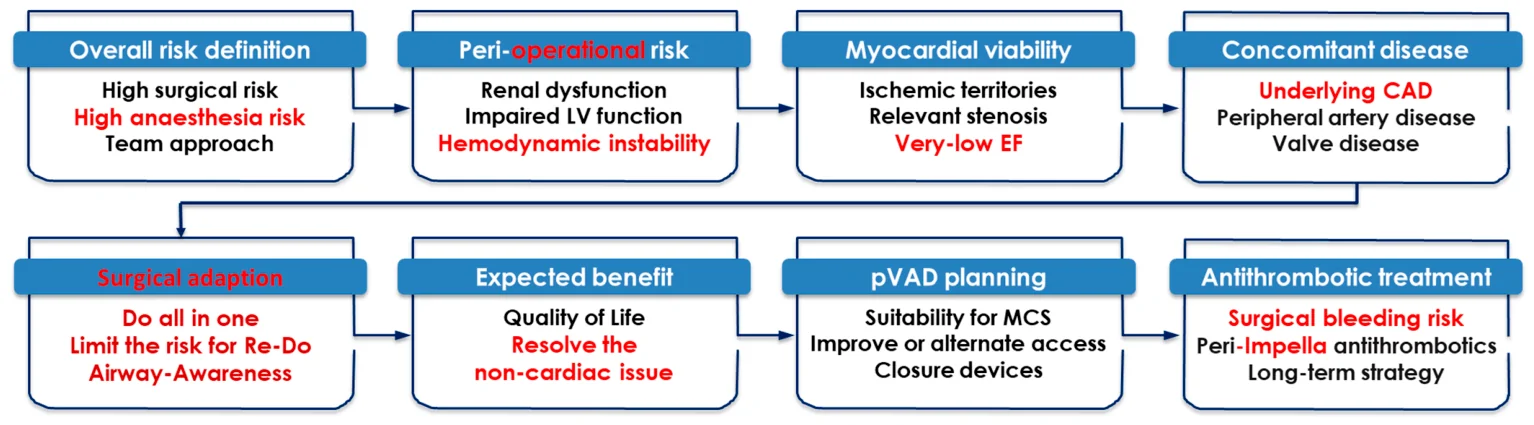
Stepwise approach to the *non-cardiac surgery* indication for mAFP in patients with severe HF/LV dysfunction. The most fundamental change to conventional approaches is that the focus is not on the shortest duration of the procedure anymore, but rather on the achievement of complete resolution in a way that no further surgery is required in patients with severely compromised LV function once the initial surgery is performed in a haemodynamically protected setting. CAD—coronary artery disease; EF—ejection fraction; HF—heart failure; LV—left ventricular; MCS—mechanical circulatory support; pVAD—percutaneous ventricular assist device.

Procedural planning for protected non-cardiac surgery differs in several respects from planning for elective coronary interventions. A proposed stepwise approach is outlined in Figure 9.

As with protected VT ablation, careful preprocedural planning is essential because successful completion of surgery does not necessarily guarantee that temporary MCS can be withdrawn immediately afterwards. Anaesthesia-related myocardial depression, perioperative fluid shifts, or other postoperative complications may temporarily impair LV function and prevent weaning from mechanical support. Patient selection should therefore include an assessment of the likelihood of prolonged MCS dependence and the feasibility of potential bailout strategies, including extended temporary support in an intensive care unit.

## 3. Discussion

Despite the widespread use of MCS devices, including the different versions of mAFPs, across an increasing range of indications, robust evidence from adequately powered and well-conducted RCTs remains scarce. To date, only the DanGer-Shock trial, which investigated the early use of Impella CP in a selected population of patients with acute STEMI-related cardiogenic shock, has demonstrated a significant mortality benefit. Even in that setting, however, important questions remain unanswered, including how best to manage patients with infarct-related shock presenting with non-ST-segment elevation myocardial infarction (NSTEMI) rather than STEMI, and those who have experienced cardiac arrest before first medical contact, which was an exclusion criterion in DanGer-Shock [2]. Many shock and cardiac arrest centres have established ECMO programmes for refractory cardiac arrest, applying varying patient selection criteria [101,102,103]. Consider, for example, a 55-year-old man presenting with an anterior AMI and cardiogenic shock. If he arrives at hospital with spontaneous circulation and meets the DanGer-Shock eligibility criteria, he may receive an Impella device. However, in some hospitals, the same patient might not receive an mAFP if he had experienced cardiac arrest before ambulance arrival and received bystander cardiopulmonary resuscitation, even if spontaneous circulation had been restored following defibrillation, which is not uncommon in patients with large anterior AMI in shock [48,75]. This is despite evidence suggesting that mAFP support may counteract the haemodynamic consequences of shock in resuscitated patients [104]. Such a scenario is not uncommon in patients presenting with extensive anterior AMI and cardiogenic shock.

The decision becomes even more challenging when cardiopulmonary resuscitation is ongoing. In this setting, many hospitals may initiate VA-ECMO despite the absence of robust evidence demonstrating a clear clinical benefit for this strategy in the relevant patient population. This example illustrates the difficulty of generating the RCT evidence required to guide real-world clinical decision-making across the full spectrum of cardiogenic shock presentations. In the meantime, clinicians must rely on the best available evidence, integrate it with individual patient characteristics and clinical circumstances, and determine whether a given intervention offers a reasonable likelihood of benefit.

Protected high-risk PCI, previously regarded as the principal indication for mAFP use, has now been investigated in two RCTs that failed to demonstrate a significant benefit for their respective primary endpoints, although both trials have limitations that need to be considered when interpreting their findings. Nevertheless, mAFPs continue to be used widely in this setting. Their use should therefore be guided by individualised clinical decision-making that carefully balances the anticipated haemodynamic benefit against procedural risks, device-related complications, and the patient’s overall clinical condition.

Looking ahead, the range of potential indications for temporary MCS may continue to expand, particularly as experience grows with the use of mechanical support during selected cardiac and non-cardiac surgical procedures. However, such expansion should be accompanied by careful patient selection, appropriate institutional preparedness, and robust clinical research to establish the safety and efficacy of these strategies.

## 4. Potential Risks and Adverse Events

As MCS devices are increasingly used across a broad range of clinical indications and procedures, their potential risks and complications must be carefully considered. These include increased procedural complexity, vascular complications associated with large-bore access, including vascular injury and impaired peripheral perfusion, and potentially serious bleeding. Other complications include haemolysis, the need for renal-replacement therapy, damage to cardiac structures such as the aortic valve or papillary muscles, and arterial thromboembolic events [18]. The incidence of complications varies according to the clinical setting, the device used, the duration of support, and the patient’s underlying condition. In studies involving acute cardiogenic shock or protected high-risk PCI, access site-related bleeding requiring transfusion has been reported in up to 15% of patients, while peripheral limb ischaemia and vascular complications have each been reported in up to 5% [2,29].

These risks highlight the importance of careful patient selection, meticulous vascular access management, appropriate anticoagulation, and systematic monitoring for device-related complications. Institutional protocols should address the prevention, early recognition, and management of these events, with particular attention to bleeding, limb ischaemia, haemolysis, and renal dysfunction. The potential benefits of haemodynamic support must always be weighed against the additional risks associated with device implantation and maintenance.

## 5. Conclusions

Although positive RCT remains limited to well-defined patient populations, mAFPs are increasingly being used to provide haemodynamic support during selected elective and urgent cardiac and even non-cardiac procedures in patients with HF and severely impaired LV function. In many of these settings, clinical decisions must be individualised and should balance the anticipated haemodynamic benefit of MCS, the expected benefit of the planned procedure, the risk of haemodynamic deterioration without mechanical support, the feasibility of performing the procedure without MCS, and the patient’s expected quality of life. The possibility of device-related complications and prolonged dependence on temporary support must also be considered.

**Figure 10:**
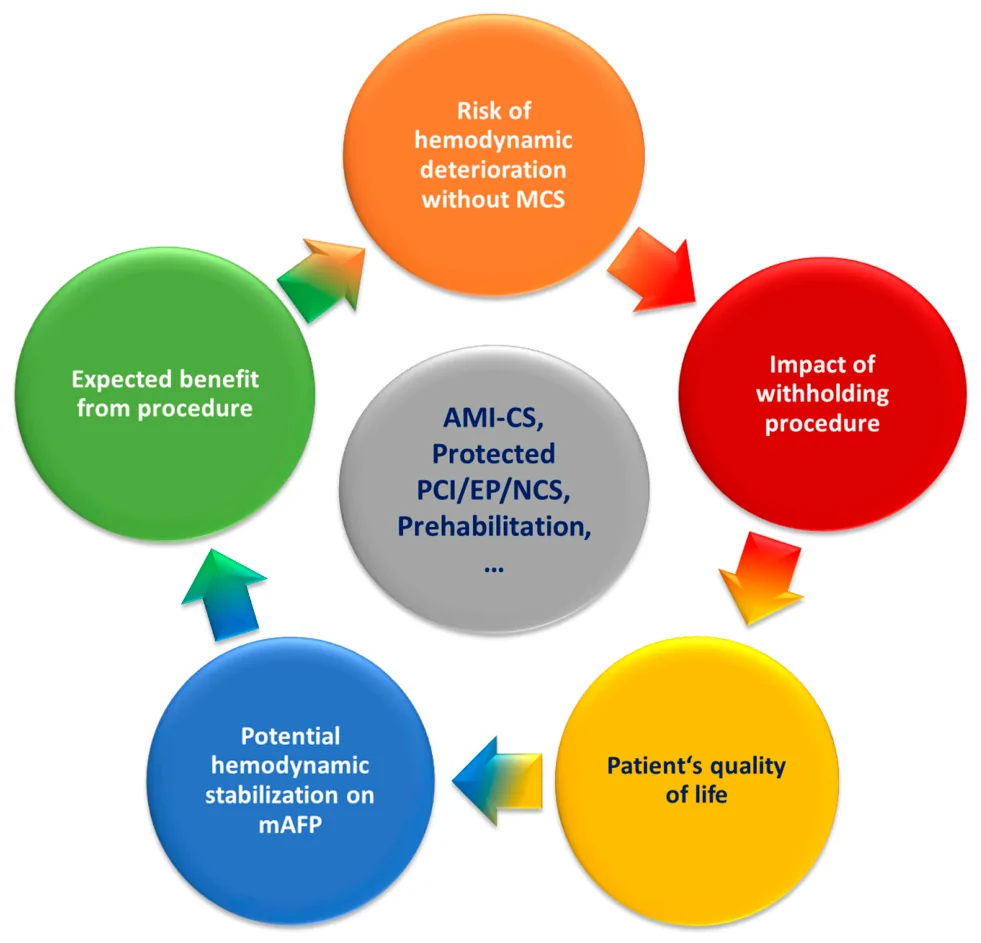
Balancing risk versus benefit of mAFP use in patients with severe LV dysfunction based on the potential effects of using or withholding mechanical circulatory support in various therapeutic procedures. AMI-CS—acute myocardial infarction-related cardiogenic shock; EP—electrophysiological ablation (e.g., of ventricular tachycardia); mAFP—micro-axial flow pump; MCS—mechanical circulatory support; NCS—non-cardiac surgery; PCI—percutaneous coronary intervention.

Regardless of the indication, structured institutional preparedness, clearly defined operational pathways, and careful procedural planning are essential. These measures may help selected patients with severely impaired LV function undergo otherwise high-risk procedures more safely and reduce the likelihood of peri-procedural haemodynamic compromise (Figure 10). However, broader adoption of mAFP support should be accompanied by continued evaluation of its safety, efficacy, and clinical outcomes across different indications.

## Figures and Tables

**Table 1:** Microaxial flow pumps (mAFP), which are/were tested in randomised controlled trials.

Device	Access/Size	Support	Classic Indication
Impella 2.5 ^a^ (not available anymore)	10F	2.5 L/min	Protected PCI
	femoral		
Impella CP ^a^	14F	3.5 L/min	AMI-CS
	femoral		
Impella 5.5 ^a^	23F	5.5 L/min	Advanced HF protected cardiac surgery
	subclavian		
Supira pVAD ^b^	10F	5.5 L/min	Protected PCI
	femoral		

^a^ Manufactured by Abiomed, Danvers, MA; ^b^ manufactured by Supira Medical, Los Gatos, CA. AMI-CS—acute myocardial infarction-related cardiogenic shock; HF—heart failure; PCI—percutaneous coronary intervention; pVAD – percutaneous ventricular assist device.

## Data Availability

No new data were created or analyzed in this study. Data sharing is not applicable to this article.

## Abbreviations

The following abbreviations are used in this manuscript:

AMI	Acute myocardial infarction
CAD	Coronary artery disease
CHF	Congestive heart failure
CS	Cardiogenic shock
CTO	Chronic total occlusion
EDPVR	End-diastolic pressure–volume relation
ESPVR	End-systolic pressure–volume relation
EP	Electrophysiology
HF	Heart failure
IABP	Intraortic balloon pump
ICU	Intensive care unit
LA	Left atrial
LCOS	Low cardiac output syndrome
LV	Left ventricular
LVAD	Left ventricular assist device
LVEDP	Left ventricular end-diastolic pressure
LVEF	Left ventricular ejection fraction
mAFP	Microaxial flow pump
MCS	Mechanical circulatory support
OAC	Oral anticoagulation
OMT	Optimal medical therapy
PCI	Percutaneous coronary intervention
pVAD	Percutaneous ventricular assist device
RCT	Randomised controlled trial
RV	Right ventricular
vaECMO	Veno-arterial extracorporeal membrane oxygenation
VT	Ventricular tachycardia
